# Edible caterpillars (*Gonimbrasia belina* and *Gynanisa maja*) as emerging source of nutrients and bioactive compounds

**DOI:** 10.1016/j.fufo.2024.100478

**Published:** 2024-12

**Authors:** Chrysantus M. Tanga, Brian O. Ochieng, Dennis Beesigamukama, Changeh J. Ghemoh, Cynthia Mudalungu, Xavier Cheseto, Isaac M. Osuga, Sevgan Subramanian, Segenet Kelemu

**Affiliations:** a International Centre of Insect Physiology and Ecology, P.O. Box 30772 – 00100, Nairobi, Kenya; b Department of Animal Science, Jomo Kenyatta University of Agriculture and Technology, P.O. Box 62000, Nairobi 00200, Kenya; c International Centre for Tropical Agriculture (CIAT), P.O. Box 823 – 00621, Nairobi, Kenya

**Keywords:** Edible caterpillars, Active ingredients, Nutrition, Health benefits, Pharmacological functions

## Abstract

Globally, despite the commercial and cultural importance of edible caterpillars (*Gynanisa maja* and *Gonimbrasia belina*), comprehensive information on their dietary and therapeutic benefits has not been fully explored. The study was aimed at evaluating the nutritional composition and bioactive compounds profile of two important edible caterpillars (*Gonimbrasia belina* and *Gynanisa maja*) in Africa. Our results demonstrated that *G. belina* and *G. maja* are capable of amplifying host plant protein (17 %) by 4.3-folds [72.8 %] and 3.6-folds [61.3 %], respectively. Lysine (32.6 mg/g), methionine (12.5–27.1 mg/g) and valine (7.7–25.4 mg/g) value were significantly harnessed. Substantial amounts of ω−3 fatty acids (methyl 5Z,8Z,11Z,14Z,17Z-eicosapentaenoate and methyl 9Z,12Z,15Z-octadecatrienoate), minerals [iron (3.6–21.2 mg/100 g), zinc (7.1–17.3 mg/100 g), calcium (55.1–60.4 mg/100 g)] and vitamins [retinol (0.02–0.03 mg/kg), γ tocopherol (0.05–0.07 mg/kg) and α-tocopherol (1.2 mg/kg)] were detected. Important phytochemical [flavonoids: rutin (7.8–20.4 ng/g), quercetin (7.2–9.0 ng/g), luteolin (7.4–7.5 ng/g), apigenin (20.8–28.6 ng/g) and kaempferol (3.3–6.5 ng/g)] and phytosterols [stigmasta-3,5-diene, campesterol and sitosterol] were identified. These findings suggests that both edible caterpillars contain sufficient nutrients and therapeutic compounds, which when integrated into human food products, either whole, in-part, or processed will greatly contribute towards countering nutritional insecurity and improve livelihoods of people in many countries.

## Highlights

• Globally, saturniid caterpillars [e.g., *G. belina* and *G. maja*] makeup 27.5% of caterpillars consumed.
• In every 100 grams of dried *G. belina* and *G. maja*, 73 and 61 grams constitute proteins, respectively.
• *G. belina* and *G. maja* are rich in lysine, methionine, omega-3 fatty acids, iron, zinc, calcium and vitamins.
• The presence of flavonoids and phytosterols unravel additional health benefits of *G. belina* and *G. maja*.
• *G. belina* and *G. maja* should be considered as functional ingredients in efforts to overcome food insecurity.

## Graphical abstract

**Figure:**
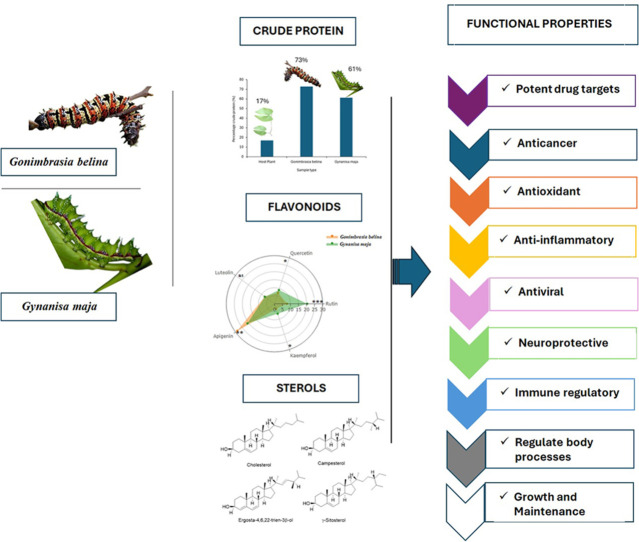

## 1 Introduction

The exponential booming of the world population has exacerbated food insecurity with food demand projected to increase by 70 % in 2050. The food situation still remains the most important impediments to both economic progress and human welfare, evidenced by the projected exponential rise in food insecurity, shattering the Sustainable Development Goals (SDG) target of eradicating all forms of malnutrition by 2030 (FAO et al., 2020). This has prompted the need to explore and exploit other non-conventional measures of combating food and nutritional insecurity among the economically marginal rural-urban fringes and rural populations, as well as affluent consumers (Kelemu et al., 2015). In this regard, the Food and Agricultural Organization (FAO) advocated for popularization of edible insect species for food and feed to enhance efforts that positively impact our current food systems (van Huis, 2015; van Huis et al., 2013).

Entomophagy is part of many cultural heritage and is transcending, from countries known for this regular practice like Africa but also in Latin America, Asia, Oceania and Europe (Glover and Sexton, 2015; Toti et al., 2020). In addition to providing food and feed for the world's expanding populace, edible insects are increasingly being acknowledged as possible sources of novel ingredients and medicinal compounds of therapeutic potential (Aiello et al., 2023; Kelemu et al., 2015; van Huis, 2012). Among these edible insect species, caterpillars especially from Africa have been reported to be nutrient-dense featuring reasonable amounts of essential nutrients. In Congo, *Imbrasia obscura* was reported to be rich in proteins (72 %), amino acids: lysine (3.3 g/100 g), methionine (1.1 g/100 g) and threonine (2.9 g/100 g), minerals: calcium (0.1 %) and zinc (154 mg/kg), and ω−3 linolenic acid (41.1 %) (Mabossy-Mobouna et al., 2018). Likewise, nutritional characterization of other edible caterpillars (*Aegocera rectilinea, Epidonta* sp and *Imbrasia truncata*) by Numbi et al. (2024) revealed protein (42.3–53.8 %), ω−3 linolenic acid (12–32 %), iron (84–362 mg/kg) and zinc (82–150 mg/kg). In North Angola, *Imbrasia epimethea* was reportedly endowed with proteins (73.1 %), lysine (4.0–5.0 g/100 g), methionine (1.2 g/100 g), threonine (3.3–3.4 g/100 g) and higher proportion of unsaturated fatty acids (59.5–68.1 %) (Lautenschläger et al., 2017). Elsewhere in Cameroon, Mba et al. (2019) reported protein (62.9–68.6 %), lysine (1.4–1.5 g/100 g), methionine (0.3–0.4 g/100 g), threonine (1.0–1.2 g/100 g), linolenic acid (1.9–2.2 g/100 g) and significant amounts of vitamin E in edible caterpillars *Imbrasia truncata* and *Imbrasia epimethea*. The rich nutritional profiles have led to the valorization of caterpillars in Africa with local communities resorting to value addition through processing to promote food security, mitigate seasonality effects and sustain the established related enterprises for incentivization (Baiyegunhi et al., 2016; Lautenschläger et al., 2017; Makhado et al., 2014).

Mopane worms, *Gynanisa maja* L. and *Gonimbrasia belina* L., are endemic to Zimbabwe, Angola, northern Namibia, Mozambique, Botswana, South Africa, Zambia, Malawi and Democratic Republic of Congo (Makhado et al., 2012). They are highly polyphagous and widely forage preferentially on mopane trees *Colophospermum mopane* (Baiyegunhi et al., 2016b), which forms almost monospecific stands over large tracts of clay-rich soils within an altitudinal range of 300–1000 m (Bara et al., 2022). The caterpillars also feed on other shrubs and tree species in areas where mopane trees are non-existent (Nantanga and Amakali, 2020). Outbreak densities of both caterpillars do occur on *Colophospermum mopane* (Mopane Bushveld) throughout their distributional range (Stack et al., 2003) and such herbivory relationships affect the nutritional constituents of each insect species. Seasonal availability of these caterpillars coincides with the rainy periods, which is associated with increased consumption at household levels across the countries (Stack et al., 2003). In the past, communities have harvested caterpillars for subsistence, but substantial nutritional impact on both rural and urban diets as well as generation of revenues for the rural dwellers has been reported (Baiyegunhi et al., 2016a). The advent of heightened demand and supply of edible caterpillar-integrated products coupled with the diversity and abundance has raised awareness of their economic relevance consequently earning the insects top priority accords in certain countries’ national development plans (Sekonya et al., 2020; Makhado et al., 2014). Trading on caterpillars remains a substantially lucrative enterprise in the Republic of South Africa with an estimated annual sale of 1600 tons of traditionally prepared and dried caterpillars worth between US$ 39–59 million with less privileged rural women actively involved in the production process garnering about 40 % of the revenue accrued (Baiyegunhi et al., 2016b; De Foliart, 1990; Makhado et al., 2014). Similarly, in Botswana's involvement in the mopane worm trade industry is reported to worth approximately between US$ 3.3 – 8 million in a good year and supports the livelihood of over 10,000 people (Sekonya et al., 2020; Stack et al., 2003). In Cameroon, Ngute et al. (2020) reported that an estimated 69.5 tons of edible caterpillars worth US$ 163,565 are marketed annually. Generally, the member states constituting the Southern African Development Community (SADC) wildly experience uneven rainfall patterns, which results into erratic upsurge of the Mopane caterpillars which are unpredictable in nature (Illgner and Nel, 2000). Thus, often, there are interruption of supplies and fluctuation in the prices which ultimately yield undesirable outcomes to risk inflicted and poor farmers (Ghazoul, 2006).

Despite the economic importance of both caterpillar species, much of the research on the mopane worms has largely focused on protein, fat and vitamins (Madibela et al., 2009; Moyo et al., 2019; Kwiri et al., 2020; Siulapwa et al., 2012) with a few studies unravelling phenolic acids, flavonoids and terpenes as the bioactive components of edible caterpillars of the genius *Cinabra, Imbrasia* and *Gonimbrasia* (Kapepula et al., 2023). A team of researchers at the International Centre of Insect Physiology and Ecology (*icipe*), Nairobi, Kenya, are now exploring a new dimension to develop low-cost technologies for mass production of these caterpillars for use as emerging food ingredients to bolster food security in Africa and beyond. Although, it has been documented that food plants and their associated edible insects do contain important phytosterols, which are highly beneficial to human health as cholesterol-lowering agent (Sabolová et al., 2016). Notwithstanding the economic significance of mopane worms, to the best of our knowledge, there is lack of information regarding the complete nutrient profile of these species. Therefore, this study seeks to proximately and spectrometrically profile the nutritional and phytochemical composition of *G. belina* and *G. maja*. The study further sought to identify potential therapeutic compounds present in the mopane worm species and the role of their food plant paying special attention to free sterols and their metabolites.

## 2 Methods

### 2.1 Sampling site and harvesting of caterpillars from the wilderness

**Fig. 1:**
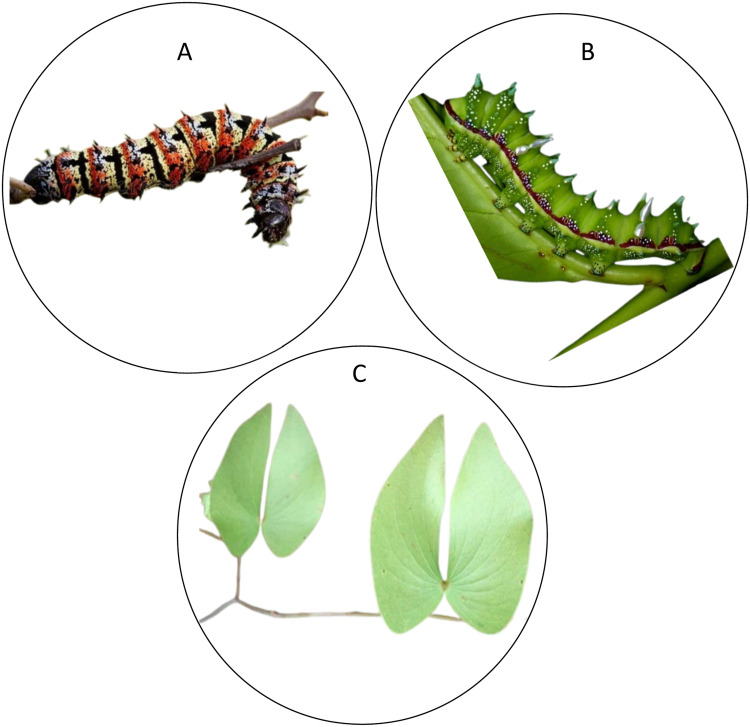
Edible caterpillars [*Gonimbrasia belina* (A) and *Gynanisa maja* (B)] and their suitable host plants (C) collected from Botswana.

Samples of *G. belina* [Fig. 1A] and *G. maja* [Fig. 1B] were acquired from Matsitama village (approximately 21° 01′ 12.00″ S, 26° 41′ 3.59″ E; 1049.73 m above sea level) located 100 km west of Francistown, Central District of Botswana. The adult stage of the two caterpillars were identified to species level as *Gonimbrasia belina* (Westwood, 1894) and *Gynanisa maja* (Klug, 1836) according to the available taxonomic keys (Kitching et al., 2018; Pinhey, 1972). The larvae of *G. belina* was morphologically identified by their approximate length and diameter of 80 mm and 10 mm, respectively, covered in dense black speckles, with shades of yellow and bluish-grey with some featuring red speckles, and possession of six short, pointed spines on each segment covered in fine white hairs (Ditlhogo et al., 1996). On the other hand, the larvae of *G. maja* was identified as green worm (Langley et al., 2020). The Mopane Bushveld is managed by the Botswana's Department of Forestry and Range Resources domiciled under the Ministry of Environment, Wildlife and Tourism, which is mandated to manage forest conservation and activities related to harvesting of these caterpillars. Therefore, only licensed individuals are legally permitted to collect non-timber and harvest timber products from forests within the country. Acquisition of the mopane worms intended for this study was therefore facilitated by a permit issued from the Ministry of Environment, Wildlife and Tourism. The edible caterpillars are widely utilized by the local communities living at the periphery of forest and beyond (Ghazoul, 2006). On the flip side, in Botswana, much of the mopane belt sits on communally-owned lands where the established customary laws inspires the local dwellers and inhabitants to capitalize on the ‘free forest resource’, especially at the time of the year coinciding with the planting season when staple stocks are often depleted (Stack et al., 2003). Therefore, this study did not embody experimentation on protected or endangered species neither did it peril other wild animals in the forest.

In Matsitama village, *G. belina* and *G. maja* feed almost exclusively and preferentially on mature leaves as opposed to tender leaves of mopane trees, *Colophospermum mopane*, which forms almost monospecific stands over large tracts of clay-rich soils. However, both species can be found feeding equally at all levels of the plant with minimal competition amongst each other (Mughogho and Munthali, 1995).

The mopane worms were harvested at the 5th instar stage of maturity, which coincides with two harvesting periods in the country (Makhado et al., 2014). Harvesting of mopane worms was conducted over a 5-hectare of communal grazing land clearly marked into 5 plots using standard traditional methods, which is increasingly becoming a routine phenomenon not only exercised by the rural poor people but also actively undertaken by people from all the other social structures involved (Stack et al., 2003). In each plot (1 hectare), 10 *C. mopane* trees hosting *G. belina* and *G. maja* were randomly selected and marked with a purple ribbon. On each tree, 5 samples of each caterpillar species were hand-picked directly from the foliage while still feeding within a surface area of 1-square meter (1 m^2^) made of wooden frame. The samples of each caterpillar species were placed separately into perforated bags to allow for ventilation to mimic the traditional post-harvest practices of keeping the insects alive until processing. In the laboratory, the caterpillars were thoroughly processed by degutting and sun drying for 2 days following the method described by Toms and Thangwana (2005). A total of 100 *C. mopane* trees were sampled in the two harvesting seasons (50 trees on the 15th of December 2015 and additional 50 on the 15th of April 2016). During the survey, we made sure no host plant was sampled twice within the same plot. The harvested caterpillars were then pooled and concocted into a composite sample weighing 6.93 kg for each caterpillar species. Thereafter, the caterpillar samples were packed in polyethylene zipper bags and transported to the laboratories at the International Center of Insect Physiology and Ecology (*icipe*), Nairobi, Kenya for analysis.

### 2.2 Plant material

In each plot, one hundred (100) mature leaf samples (50 leaves at heights between 0 – 3 m and 50 between 3 – 10 m) [Fig. 1C] were harvested within the same 1 m^2^ surface area of each tree where the caterpillars were collected. The harvested leaves were transferred into perforated polyethylene bags. During sampling, leaf samples were collected randomly from different plants. The leaf samples were then dried in the sun for 2 days and processed into powder form, and packed in sterilized zipper bags and transported to the laboratories at *icipe*, Nairobi, Kenya for analysis. The harvested leaf samples were then lumped into a single composite sample of 3.76 kg for the entire two harvesting seasons. We made sure that during the entire survey, no food plant was sampled twice within the same plot during the two field trips.

### 2.3 Chemical assessment

#### 2.3.1 Proximate determination

The proximate components were measured according to AOAC (2016). Moisture, dry matter, crude fat, protein, total ash and fibre were determined on mopane tree leaves whereas acid detergent fibre (ADF), protein, crude fat and neutral detergent fibre (NDF) were determined on the mopane caterpillars. Moisture and dry matter were assessed by 105 ℃ drying for 3 h in an oven. Kjeldahl analysis of nitrogen content and a subsequent 6.25-conversion factor to protein considered for protein estimation. Fat was estimated in a Soxhlet apparatus by extraction using petroleum ether. Total ash was estimated by kindling of sample for 3 h at 550 ℃ in a muffle furnace. Neutral detergent and acid detergent fibres were analyzed by reagent digestion as described by Van Soest et al. (1991) in a fibre analyzer. The organic matter was computationally determined by difference.

#### 2.3.2 Amino acid analysis

At 110 ℃ for 24 h, 100 mg of each sample was subjected to anaerobic hydrolysis using 1.5 mL of 6 N HCl in hermetically sealed glass tubes filled with nitrogen. The hydrolysates were subsequently dried under vacuum, reconstituted with 1 mL mixture of acetonitrile and 0.01 % formic acid (5:95), agitated by vortexing and sonication for 30 s and 30 min, respectively and then centrifuged for 15 min at 14,000 rpm. The supernatants were filtered with a 0.22 μ m microporous filter membrane and analyzed (0.2 μL) using LC-MS. The chromatographic analysis was achieved on an Agilent system 1100 series (MA, USA) using ZORBAX SB-C18, 4.6 × 250 mm, 3.5μm column, operated at 40 °C. The gradient elution program, instrument operating conditions and quantification of the individual amino acids adhered to protocols outlined by Cheseto et al. (2017). The determinations were repeated three times using different batch of samples.

#### 2.3.3 Fatty acids profiling

The lipid profiles of the caterpillars (100 mg each), were determined as fatty acid methyl esters (FAMEs) in a GC–MS according to modified methods and instruments operating conditions previously adopted (Cheseto et al., 2015, 2020). Briefly, samples were methyl esterified with 1 mL of methanolic sodium methoxide solution. Upon vortexing, sonication and conditioning in 70 ℃-water bath for 1 min, 10 min and 1 h, respectively, 100 *µ*L of distilled deionized water was introduced to each sample to terminate the reaction for 1min. The resultant FAMEs were extracted with 1 mL of hexane through a centrifugation for 5 min at 14,000 rpm. Anhydrous sodium sulphate-dried supernatants were filtered and the filtrate (1.0 *µ*L) analyzed by GC–MS on a 7890A gas chromatograph fitted with a (5 %-phenyl)-methylpolysiloxane (HP5 MS) low bleed capillary column (30 *m* × 0.25 mm i.d., 0.25 *µ*m; J&W, Folsom, CA, USA) and linked to a 5975 C mass selective detector (Agilent Technologies Inc.,Santa Clara, CA, USA). The analysis was conducted in triplicates.

#### 2.3.4 Mineral composition

The mineral contents were assessed following methods adopted by Ochieng et al. (2022). In brief, 0.5 g of ground caterpillar were wet-ashed by digesting with 8.0 mL of 68–69 % w/v nitric acid and 2 mL of 30 % w/w hydrogen peroxide on a heated block digester in a fume hood. The contents of the digest were analyzed in an Inductively coupled plasma optical emission spectrometry (ICP-OES) measurements (Optima 2100TMDV ICP-OES, Perkin Elmer Massachusetts, USA). A standard curve (R^2^ = 0.999), generated by also analyzing ICP-OES mix standard CatNo.43843 (Sigma-Aldrich, USA), was adopted for external quantification of the mineral elements under study.

#### 2.3.5 Vitamin determination

The approach described by Thermo Fisher Scientific (2010) was used to determine water soluble vitamins. Exactly 0.1 g of caterpillar samples were suspended in 25 mL of deionized water, sonically agitated for 15 mins and membrane filtered through 0.22 μm aperture polycarbonate filter (Millipore; Billerica, MA, USA) into 1.5 mL vials. The filtrates were analyzed at 30 °C in a Liquid chromatographic system with Diode Array Detector (LC-30AC with Nexera column oven CTO-30A, Shimadzu, Tokyo, Japan) fitted with a Phenomenex C18 Column Synergi 100 × 3.00 mm, 2.6 µm polar (Phenomenex, Torrance, CA, USA) at. A gradient elution comprising of two solvents was set as follows; A: 25 mM phosphate buffer and B: 7:3 v/v Acetonitrile-Mobile phase A made up the mobile phase. Over 12 min, chromatographic separations were achieved at a flow rate of 0.4 mLmin^−1^. Four standards (2, 5, 10 and 15µg/mL) formulated from stock solutions of 1.0mg/mL were also analyzed in the HPLC to generate calibration curves for external quantification.

The fat-soluble vitamins were determined following procedures outlined by Bhatnagar et al. (2015). In a homogenizer, 500 mg of each caterpillar flour was blended with 6 mL of ethanol premixed with 0.1 % of butylated hydroxy toluene. The homogenate was then mixed with 120 µL of aqueous potassium hydroxide (80 %, w/v), vortexed for a minute, incubated at 80 °C for 5 min and later ice-cooled. Four-milliliters of deionized water was then transferred to the mixture and vortexed again for 1 min. Extraction of the fat-soluble vitamins was accomplished by addition of 5 mL HPLC-grade hexane and subsequently centrifuged for 5 min at 3000 rpm. The supernatants were hexane extracted, pipetted carefully into different test tubes and the upper phases pooled. The extracted mixtures were dried in vacuo and the resulting residues redissolved in 1 mL mixture of tetrahydrofuran and HPLC grade methanol (15:85 v/v), vortexed and sonicated for 1 min and 30 s, respective, and finally filtered into 1.5 mL vials. The samples (10 μL) were analyzed by a Reverse-phase HPLC (Shimadzu Nexera UPLC system) fitted with YMC C30, carotenoid column (3µm, 150×3.0 mm, YMC Wilmington, NC) and linked to SPD -M2A detector at a flow rate of 0.4 mL/min. All determinations were done in three replicates.

#### 2.3.6 Flavonoids determination

Dry caterpillars were milled in liquid nitrogen and then suspended in 10 mL of 80 % (V/V) aqueous methanol. Extraction of the flavonoids were enhanced by agitation through vortexing and sonication for 1 min and 1 h, respectively. The pellets were then re-extracted twice with subsequent pooling together of the extracts. The extracts were subjected to a 15-min centrifugation at 4200 rpm, the supernatants filtered and subsequently chromatographically separated on Agilent 1260 Infinity HPLC system (Agilent Technologies, Palo Alto, CA) fitted with a 40 °C-operated ZORBAX SB-C18, 4.6 × 250 mm, 3.5μm column and coupled to an Agilent 6120 mass detector MS with a single quadrupole analyzer (Agilent Technologies, Palo Alto, CA). The elution gradient system and the instrument operating parameters were set as outlined by Cheseto et al. (2024). The analysis was repeated three times using different batches of caterpillar flours samples.

#### 2.3.7 Sterol analysis

About 10 g of each of dried caterpillar powder was extracted with aqueous methanol (80 %, v/v) and evaporated under vacuum atmosphere. The residual aqueous phase was mixed with equal volume of distilled water. Furtherance to this, the mixture was partitioned (liquid-liquid extraction) with another equal volume of hexane to extract the layer of fatty acids. The remaining lower layer was subsequently re-extracted with equal volume of dichloromethane. The dichloromethane extract was concentrated under vacuum atmosphere and reconstituted to a 100 ng/μL. The sample (1.0 µL) was analyzed by GC–MS on a 7890A gas chromatograph fitted with a (5 %-phenyl)-methylpolysiloxane (HP5 MS) low bleed capillary column (30 *m* × 0.25 mm i.d., 0.25 *µ*m; J&W, Folsom, CA, USA) and linked to a 5975 C mass selective detector (Agilent Technologies Inc.,Santa Clara, CA, USA). The instrument operating conditions and the sterol identification procedures described by Mudalungu et al. (2023) were applied. The determinations were done in triplicates.

### 2.4 Statistical analysis

R software version 4.0.2 for windows (R Core Team, 2020) was used for data analysis at 5 % significant level. Bartlett test (*p* < 0.05) was used to explore the homogeneity of the variances of the data groups. Student's *t*-test was adopted for differentiation of homoscedastic data whereas a Welch *t*-test was applied to compare data sets with unequal variances from *Gonimbrasia belina and Gynanisa maja* on proximate values, fatty acids, amino acids, minerals, flavonoids and vitamins.

## 3 Results

### 3.1 Nutrient composition of mopane tree leaves and mopane caterpillars

**Fig. 2:**
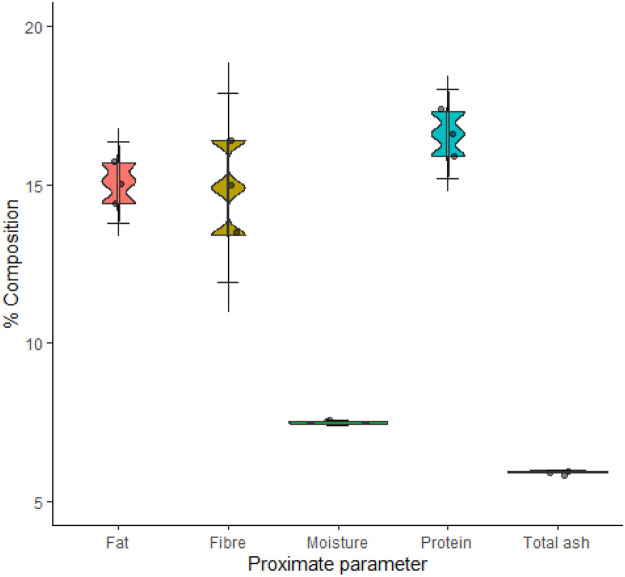
Proximate composition (dry weight) of mopane tree leaves.

Protein (16.6 %), fat (15.1 %) and fibre (14.9 %) were the dominant macromolecular components of the mopane tree leaves (Fig. 2).

There were significant differences (*p* < 0.05) in protein, organic matter and neutral detergent fibre between *G. belina* and *G. maja* (Table 1). *G. belina* exhibited significantly higher levels of organic matter, protein and neutral detergent fibre.

**Table 1:** Proximate values (% dry weight) of the caterpillars (mean ± SE).

Parameter	*G. belina*	*G. maja*	t-value	df	p-value
Dry matter	91.6 ± 0.80	89.8 ± 0.17	2.1606	4	0.097
Crude ash	3.9 ± 0.19	9.7 ± 0.06	−29.147	4	8.25E-06
Organic matter	96.2 ± 0.19	90.3 ± 0.06	29.147	4	8.25E-06
Crude protein	72.8 ± 0.12	61.3 ± 0.13	64.249	4	3.52E-07
Crude fat	17.5 ± 0.20	16.4 ± 0.41	2.361	4	7.76E-02
Neutral detergent fibre	21.2 ± 0.13	24.8 ± 0.36	−9.3348	4	2.27E-01
Acid detergent fibre	14.0 ± 0.12	10.9 ± 0.27	10.369	4	4.88E-04

### 3.2 Amino acids profile

Twelve amino acids were found in the caterpillars, with essential amino acids (EAA) and non-essential amino acids (NEAA) contributing seven and five parts, respectively (Table 2). Significant differences (*p* < 0.05) occurred in the concentration of all the amino acids between the two caterpillars. *Gynanisa maja* exhibited higher levels of methionine, isoleucine, histidine, phenylalanine and lysine whereas *G. imbrasia* expressed higher leucine and valine levels.

**Table 2:** Amino acids levels (mg/g dry weight) of the caterpillars (mean ± SE).

Amino acid	*G. belina*	*G. maja*	t-value	df	p-value
Glutamate	8.5 ± 0.15	7.2 ± 0.05	8.30	4	0.001
Arginine	68.8 ± 0.10	56.2 ± 0.10	91.14	4	8.69E-08
Isoleucine	32.0 ± 0.21	49.5 ± 0.12	−73.17	4	2.09E-07
Leucine	70.5 ± 0.13	11.5 ± 0.03	449.74	4	1.47E-10
Proline	72.0 ± 0.19	77.6 ± 0.10	−25.24	4	1.46E-05
Valine	25.5 ± 0.11	7.7 ± 0.07	136.72	4	1.72E-08
Methionine	12.5 ± 0.07	27.1 ± 0.13	−99.8	4	6.05E-08
Hydroxyproline	34.9 ± 0.03	12.8 ± 0.24	91.27	2.05	9.61E-05
Tyrosine	101.3 ± 0.31	93.0 ± 0.15	24.2	4	1.73E-05
Histidine	18.9 ± 0.07	24.8 ± 0.10	−48.28	4	1.10E-06
Lysine	32.6 ± 0.25	45.3 ± 0.29	−33.12	4	4.96E-06
Phenylalanine	76.0 ± 0.18	107.3 ± 0.26	−97.73	4	6.57E-08

### 3.3 The fatty acids constituents of mopane worm

The spectra of fatty acids from the two caterpillars are indicated in Table 3. For the *G. belina*, SFAs, MUFAs and PUFAs contributed 82.0 %, 12.5 % and 5.5 %, respectively, of the total fatty acids while for the *G. maja*, SFAs, MUFAs and PUFAs 80.2 %, 11.1 % and 1.0 % respectively of all the profiles. Among the PUFAs, significant differences (*p* < 0.05) occurred between *G. belina* and *G. maja* with regards to the concentrations of methyl 9Z,11E,13E-octadecatrienoate and methyl 5Z,8Z,11Z,14Z-eicosatetraenoate. On the other hand, no significant difference was observed for methyl 9Z,12Z,15Z-octadecatrienoate and methyl 5Z,8Z,11Z,14Z,17Z-eicosapentaenoate. The Σn-6/n-3 ratio was 0.25 and 0.92 while the ΣPUFA/SFA was 0.07 and 0.02 for *G. belina* and *G. maja* respectively.

**Table 3:** Fatty acid composition (µg/g dry weight) the mopane worms.

tR (min)	Compound	*G. belina*	*G. maja*	t-value	df	p-value
13.38	Methyl octanoate	0.2 ± 0.01	0.6 ± 0.10	−3.63	2.06	0.065
17.79	Methyl 2,6-dimethyltridecanoate	0.1 ± 0.01	0.3 ± 0.06	−2.20	4	0.145
19.24	Methyl dodecanoate	6.1 ± 0.72	2.4 ± 0.12	4.14	4	0.048
20.39	Methyl tridecanoate	55.5 ± 11.08	0.5 ± 0.03	4.05	2	0.056
21.06	Methyl 12-methyltridecanoate	5.5 ± 0.41	0.6 ± 0.03	9.60	2.02	0.010
21.25	Methyl tetradecanoate	7.1 ± 0.75	3.7 ± 0.62	2.81	4	0.050
23.35	Methyl hexadecanoate	91.5 ± 10.51	189.4 ± 5.70	−6.69	4	0.006
25.26	Methyl octadecanoate	67.3 ± 7.93	78.2 ± 3.39	−1.04	4	0.383
26.41	Methyl nonadecanoate	2.5 ± 0.26	17.2 ± 0.99	−11.71	4	0.004
27.02	Methyl eicosanoate	0.1 ± 0.02	1.4 ± 0.24	−4.36	2.04	0.047
27.26	Methyl 18-methylnonadecanoate	4.9 ± 1.44	52.3 ± 3.64	−9.89	4	0.004
28.07	Methyl heneicosanoate	10.9 ± 1.29	16.4 ± 3.31	−1.27	4	0.305
28.87	Methyl docosanoate	3.2 ± 0.41	9.7 ± 0.34	−9.92	4	0.001
29.63	Methyl tricosanoate	2.6 ± 0.23	7.0 ± 0.32	−9.40	4	0.001
30.45	Methyl tetracosanoate	5.2 ± 1.86	6.2 ± 0.82	−0.38	4	0.734
32.48	Methyl hexacosanoate	1.6 ± 0.57	1.6 ± 0.22	0.04	4	0.971
	**ΣSFA**	259.2 ± 7.87	335.2 ± 11.89	−7.30	3.92	0.002
21.20	Methyl (5Z)-dodecenoate	3.1 ± 0.53	2.0 ± 0.34	1.33	4	0.267
21.25	Methyl (9Z)-tetradecenoate	5.2 ± 0.25	7.4 ± 0.36	−4.09	4	0.019
25.03	9E-Octadecenoate	1.1 ± 0.05	4.5 ± 0.22	−12.18	4	0.005
26.21	Methyl 10-Nonadecenoate	2.1 ± 0.25	25.2 ± 1.97	−9.52	2.06	0.010
27.05	Methyl 11Z-Eicosenoate	28.8 ± 7.29	14.3 ± 1.70	1.58	4	0.242
	**ΣMUFA**	40.3 ± 7.17	53.4 ± 2.68	−1.40	2.55	0.270
25.80	Methyl 9Z,12Z,15Z-Octadecatrienoate (ALA)	2.9 ± 0.22	4.1 ± 0.28	−2.68	4	0.058
25.83	Methyl 9Z,11E,13E-octadecatrienoate	2.7 ± 0.32	24.7 ± 1.23	−14.10	4	0.003
26.74	Methyl 5Z,8Z,11Z,14Z-Eicosatetraenoate	3.0 ± 0.35	8.3 ± 0.95	−4.27	4	0.033
26.80	Methyl 5Z,8Z,11Z,14Z,17Z-Eicosapentaenoate (EPA)	9.0 ± 3.27	4.9 ± 0.22	1.02	2.02	0.415
	**ΣPUFA**	22.5 ± 4.73	94.4 ± 4.49	−5.74	2.30	0.021
	Σ n-6 PUFA	3.0 ± 0.35	8.3 ± 0.95	−4.27	4	0.033
	Σ n-3 PUFA	11.96 ± 3.77	9.04 ± 0.10	0.77	2.00	0.520
	Σn-6/n-3	0.25	0.92			
	ΣPUFA/SFA	0.09	0.28			

Values are expressed as mean ± SE (Standard Error) of triplicate determinations. PUFA- polyunsaturated fatty acids; EPA-eicosapentaenoic acid; SFA-Saturated fatty acids; ALA-α-linolenic acid; MUFA- monounsaturated fatty acids; tR-Retention time.

### 3.4 Minerals profile

A total of 11 minerals were detected in the caterpillars (Table 4). Magnesium, sodium and potassium were the most pronounced minerals. The levels of iron, zinc, aluminium, manganese, sodium and potassium significantly differed (*p* < 0.05) between the two caterpillars. Higher iron, aluminium, manganese and sodium were detected in *G. belina* whereas higher zinc and potassium were detected in *G. maja*.

**Table 4:** The mineral profile (mg/100 g dry weight) of caterpillars (mean ± SE).

Minerals	*G. belina*	*G. maja*	t-value	df	p-value
Mg	140.5 ± 4.32	152.2 ± 5.26	−1.72	4	0.161
Fe	21.2 ± 1.09	3.6 ± 0.13	16.00	2.05	0.003
Zn	7.1 ± 0.36	17.3 ± 0.65	−13.73	4	<0.001
Ca	60.4 ± 0.78	55.1 ± 2.78	1.86	4	0.136
Cu	0.6 ± 0.03	0.7 ± 0.02	−0.66	4	0.548
Al	7.0 ± 0.27	4.8 ± 0.18	6.52	4	0.003
P	84.6 ± 1.80	91.5 ± 4.20	−1.50	4	0.207
Mo	0.2 ± 0.01	0.2 ± 0.01	2.69	4	0.055
Mn	3.9 ± 0.17	0.8 ± 0.04	17.84	4	<0.001
Na	2443.5 ± 107.28	1946.6 ± 76.40	3.77	4	0.020
K	965.2 ± 32.21	1076.0 ± 17.18	−3.03	4	0.039

### 3.5 Vitamins content

A total of eleven vitamins were detected from the two caterpillars (Table 5). The levels of all the vitamins with the exception of α tocopherol varied significantly (*p* < 0.05) between the two edible caterpillars. The levels of retinol and γ tocopherol were higher in *G. belina* while higher vitamin C were detected in *G. maja*. The B vitamins and vitamin C were found to bethe most abundant.

**Table 5:** Vitamin concentration (mg/kg dry weight) of the caterpillars on dry matter basis (mean ± SE).

Vitamin	*G. belina*	*G. maja*	t-value	df	p-value
Vitamin C	122.8 ± 4.72	225.7 ± 3.73	−17.09	4	6.87E-05
Vitamin B1	353.6 ± 8.30	526.2 ± 4.27	−18.49	4	5.03E-05
Nicotinic acid	576.8 ± 10.31	471.1 ± 15.77	5.61	4	0.005
Vitamin B6	549.2 ± 14.27	247.3 ± 12.53	15.90	4	9.15E-05
Vitamin B5	7359.5 ± 118.86	9074.4 ± 215.67	−6.96	4	0.002
Vitamin B9	1097.3 ± 16.97	2180.5 ± 74.71	−14.14	4	1.45E-04
Vitamin B12	18.18 ± 1.11	35.51 ± 1.41	−9.66	4	0.001
Vitamin B2	32.40 ± 1.11	7.0 ± 0.24	22.44	4	2.33E-05
Retinol	0.03 ± 0.00	0.02 ± 0.00	10.22	4	0.001
γ tocopherol	0.07 ± 0.01	0.05 ± 0.00	3.32	4	0.029
α tocopherol	1.2 ± 0.09	1.2 ± 0.03	−0.16	4	0.882

### 3.6 Flavonoids

**Fig. 3:**
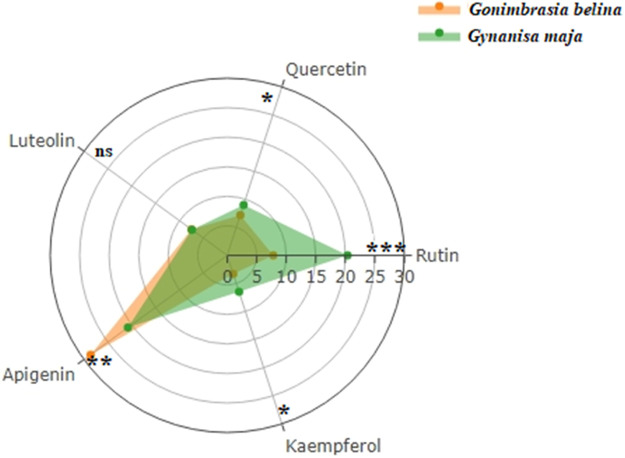
Radar chart comparing the flavonoids levels (ng/g dry weight) in G. belina and G. maja. * ns-not significant; Significant at >0.01; **Significant at >0.001; ***Significant at >0.0001;.

Five flavonoids (rutin, quercetin, luteolin, apigenin and kaemferol) were identified from the edible caterpillars (Table 6 and Fig. 3). Apigenin and rutin were the most abundant while kaempferol was the least abundant flavonoid. Significant variations were observed in all the flavonoids except luteolin. Rutin, quercetin and kaempferol were more pronounced in the *G. maja*.

**Table 6:** Concentrations (ng/g dry weight) of flavonoids in the edible caterpillars on dry matter basis (mean ± SE).

Flavonoid	*G belina*	*G maja*	t-value	df	p-value
Rutin	7.8 ± 0.08	20.4 ± 0.07	−120.40	4	2.85E-08
Quercetin	7.2 ± 0.04	9.0 ± 0.23	−7.63	4	0.002
Luteolin	7.5 ± 0.14	7.4 ± 0.06	0.67	4	0.540
Apigenin	28.6 ± 0.15	20.8 ± 0.18	33.579	4	4.69E-06
Kaempferol	3.3 ± 0.03	6.5 ± 0.08	−36.04	4	3.54E-06

### 3.7 Sterols

**Fig. 4:**
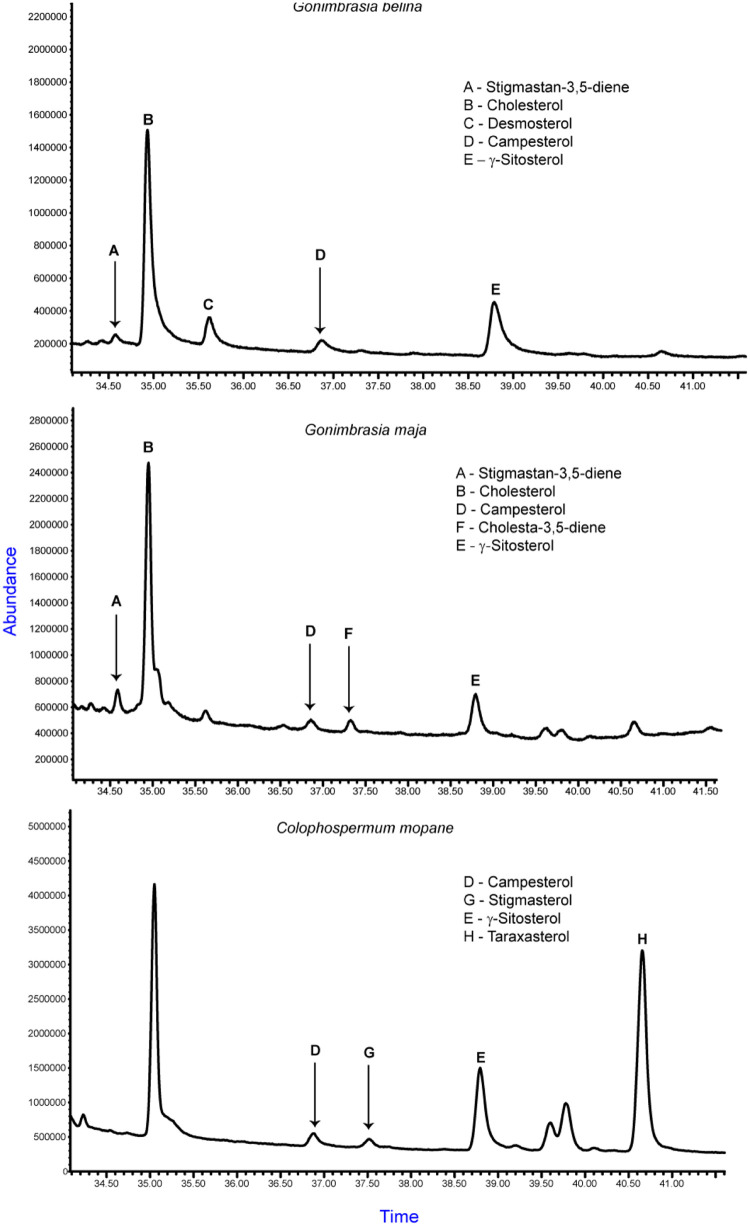
*Representative chromatograms of sterols detected from* Gynanisa maja*,* Gonimbrasia belina *and* Colophospermum mopane *tree leaves.*

Qualitative assessment indicated the presence of stigmastan-3,5-diene, desmosterol, cholesterol, sitosterol and campesterol in *G. belina*, stigmastan-3,5-diene, cholesterol, campesterol, cholesta-3,5-diene and sitosterol in *G. maja*, and β-sitosterol, stigmasterol, campesterol and taraxasterols in the mopane tree leaves ((Table 7 and Fig. 4)) following identification from GC–MS chromatogram and the respective mass spectra (supplementary information).

**Table 7:** Sterols qualitatively detected in the edible caterpillars and their dietary source.

Sample type	tR (min)	Sterol Present
Caterpillar *(Gonimbrasia belina)*	34.57	Stigmastan-3,5-diene
	34.93	Cholesterol
	35.62	Desmosterol
	36.86	Campesterol
	38.79	β-Sitosterol
Caterpillar *(Gonimbrasia maja)*	34.59	Stigmastan-3,5-diene
	34.95	Cholesterol
	36.86	Campesterol
	37.33	Cholesta-3,5-diene
	38.79	β-Sitosterol
Mopane tree leaves (*Colophospermum mopane*)	36.88	Campesterol
	37.52	Stigmasterol
	38.79	β-Sitosterol
	40.66	Taraxasterol

## 4 Discussion

The results on protein levels of studied *Colophospermum mopane* (mopane tree) leaves corroborates the mean value of 14.1 % previously reported by Lukhele and Van Ryssen (2003) and 13.45 % reported by Nunu et al. (2019). Even though little has been done on the fat content of mopane tree leaves, the considerable levels found in this study is reminiscent of the essential fatty acids previously detected in the leaves, known to significantly contribute to the browsers’ nutritional health (Makhado et al., 2016). The larval stages of both the mopane caterpillars voraciously defoliates the nutrient rich-leaves of mopane trees (Bara et al., 2022) to derive nutrients which play important roles in the biochemical processes crucial to the growth and development of the insects and other browsers (Mahmoud, 2017). Fibre influences the acceptability and digestibility of forages by potential browsers. The fibre content of the mopane leaves in the present study were less than 21.9–28.1 % reported cross-seasonally by Makhado et al. (2016). These variation might be attributable to the environmental condition, season/interannual climatic variability and growth stage of the leaves at the time of collection (Makhado et al., 2018).

The protein level of both *G. belina* and *G. maja*, though slightly higher, accords the levels of 57.0 % and 55.9 %, respectively previously reported by Siulapwa et al. (2012). These protein levels comparably supersedes those of conventional sources of meat such as veal, lamb, beef and mutton (Williams, 2007). It may be hypothesized that consumption of 100 g of these caterpillars by a 70 kg adult human would guarantee almost 2-folds the recommended daily allowance (RDA) for protein based on the FAO/WHO reference values (Lewis, 2019). Therefore, these caterpillars offer derivable nutrients requisite for counteracting protein energy malnutrition bedeviling inhabitants of developing countries (Potgieter et al., 2012). Hypothetically, the notable disparity in the protein level between *G. belina* and *G. maja* may be accredited to genetic differences in feed bioconversion or differences in chemical composition of 0–3 m occurring leaves that *G. maja* fed on and 3–10 m occurring leaves that *G. belina* fed on. Researchers have previously reiterated the abundance of fat in the lepidopterans (Hlongwane et al., 2020; Rumpold and Schlüter, 2013). Fat levels were higher than 10 % and 12.1 % reported in *G. belina* and *G. maja*, respectively (Siulapwa et al., 2012) but comparable to 15.7 % average fat computed from several caterpillars consumed in Africa (H. Womeni et al., 2009). Fats are key calorigenic components of food hence, fats from these edible caterpillars can considerably cushion against calorigenic deficiencies as well as modulate the flavour characteristics of the insects upon roasting and frying (H.M. Womeni et al., 2009). The NDF and ADF were slightly lower than the values reported by Madibela et al. (2009) on degutted *Imbrasia belina*.

Amino acid composition determines the nutrient values, particularly protein quality of food products. The amino acids profile (except for hydroxyproline) have previously been discovered in *G. belina* and reported to compare exceptionally with soybean (Kwiri et al., 2020). Methionine and lysine, which are commonly known limiting amino acids in pulses and maize, were detected in significant amounts. Further, being indispensable amino acids in child growth and development, the remarkable levels of arginine and tyrosine in the caterpillars demonstrates their suitability for child nutrition (Mba et al., 2017). This indicates that mopane worms can be utilized in supplementation of plant-predominant diets recognizable with imbalanced amino acids profiles. In this regard, local communities blend caterpillars with staple foods to prepare nutritious paps or processed before integrating into other food such as porridge to offset their nutritional inferiority and counteract child malnutrition (Solomon and Prisca, 2012). Lysine and tryptophan have been reported as the limiting amino acids in various edible insects (Udomsil et al., 2019), however in this study, appreciable amounts of lysine were detected. The variations in limiting amino acids levels is purportedly dependent on the insect diet and species (Ghosh et al., 2017).

The proportions of SFAs, MUFAs and PUFAs of the studied caterpillars were inconsistent with 19.6 % PUFAs, 34 % MUFA and 38 % SFA, recorded in *G. belina* (van Huis, 2020), 45.1 % PUFA, 5.2 % MUFA and 32.2 % SFA found in *Imbrasia epimethea* (Mabossy-mobouna et al., 2021) and 48.7 % PUFAs, 10.0 % MUFAs and 41.2 % SFAs detected in *Imbrasia truncata* (Mba et al., 2017). Such intra- and interspecific differences in total fatty acids may have emerged from variations in dietary sources, light and temperature (Oonincx and Finke, 2020). The experimental caterpillars were collected from same host trees and environmental conditions, which may explain the insignificant differences in their total fatty acid proportions. Previous studies have listed methyl 9Z,12Z,15Z-octadecatrienoate, methyl 9Z-octadecenoate, and methyl hexadecanoate and methyl octadecanoate, as the predominating PUFA, MUFA and SFAs, respectively in *G. belina* (Kwiri et al., 2020), *Imbrasia oyemensis* (Akpossan et al., 2015) and a variety of *Imbrasia* sp. (H. Womeni et al., 2009). This study found similar results on dominant SFAs but contradicting ones on MUFAs and PUFAs from the two caterpillars. Nevertheless, appreciable levels of methyl 5Z,8Z,11Z,14Z,17Z-eicosapentaenoate (EPA) and methyl 9Z,12Z,15Z-octadecatrienoate (ALA) were detected which are ω−3 fatty acids known to induce suppressive effects on the occurrence of cardiovascular disorders, cancer, inflammatory and autoimmune diseases (H. Womeni et al., 2009). On the other hand, methyl 5Z,8Z,11Z,14Z-eicosatetraenoate and methyl 9Z,12Z,15Z-octadecatrienoate are metabolically indispensable agents for growth, physiological functions and body maintenance (Akpossan et al., 2015). The PUFA/SFA ratio of *G. maja* was >0.20, which is concomitant with higher cholesterol lowering potential and cardio-friendliness (Mabossy-mobouna et al., 2021). Interestingly, the Σn-6/n-3 of both *G. belina* and *G. maja* were within the FAO ratio of 10:1 and corroborated those reported from other caterpillars (Guil-Guerrero et al., 2018). This implies that the mopane caterpillars are pro-health and wellness enhancers and is applicable in dietetic management of thrombogenic illnesses (Atowa et al., 2021; H. Womeni et al., 2009).

Minerals are integral to enzyme and protein functionalities and plays central roles in metabolic and physiological processes. The richness of caterpillars in K, Ca, Mg, Zn, P, Fe have previously been documented (Kwiri et al., 2020; Oleko, 2018; Paiko et al., 2014). In particular, the significantly higher Fe and Na in *G. belina* and high Ca in *G. maja* endorses the findings of Siulapwa et al. (2012) on the two caterpillars. The levels of these minerals however, are a function of their respective elemental concentrations in dietary sources, age and ecotype (Paiko et al., 2014). Based on the nutrient reference values published by FAO/WHO, consumption of 100 g of *G. belina* would contribute about 57.6 % Zn Recommended Dietary Intake (RDI), 123.9 % Fe RDI, 6.0 % Ca RDI, 12.1 % P RDI, 45.3 % Mg RDI, 66.7 Cu RDI and 130 % Mn RDI whereas 100 g of *G. maja* would offer approximately 190.5 % Zn RDI, 101.1 % Fe RDI, 5.5 % Ca RDI, 13.1 % P RDI, 49.1 % Mg RDI, 77.8 Cu RDI and 26.7 % Mn RDI (Lewis, 2019). K and Na are key minerals in the management of blood pressure, Ca is a crucial mineral for bone mineralization and blood coagulation, trace minerals Zn and Cu plays various physiological roles in the body (Das and Mandal, 2013). Except for vitamin B1 and C, the water and fat soluble vitamins evaluated in this study had previously been reported by Tanga et al. (2023) on a saturniid caterpillar, *Gonimbrasia cocaulti* which is closely related to the study caterpillars. Similarly, Ouma et al. (2022) detected retinol, ɣ-tocopherol and α-tocopherol in *Gonimbrasia zambesina*. Insects may be acquiring these micronutrients from plant matter they feed on, however, supporting information are still scanty (Oonincx and Finke, 2020). Consumption of 100 g of *G. belina* can provide approximately 122.8 % of vitamin C RDI, 3.8 % of vitamin A RDI and 13.3 % of vitamin E RDI whilst *G. maja* could deliver 225.7 % of vitamin C RDI, 2.5 % of vitamin A RDI and 13.3 % of vitamin E RDI (Lewis, 2019). Since animals are metabolically incapacitated to synthesize vitamins *de novo*, they rely on dietary sources to meet their body needs. Therefore, *G. belina* and *G. maja* can be exceptional dietary supply of vitamins to consumers. As such, caterpillars can be appropriate candidates for combating micronutrient-related deficiencies disconcerting over 2 billion people (FAO, 2013) particularly women and children of the peri‑urban and rural dwellers, manifesting as anemia and stunting (Roos and van Huis, 2017).

Exploration of insect phenolic compounds, particularly caterpillars, has yielded quercetin-glycosides and kaempferol-glycosides in *Rondotia menciana* and quercetin, quercetin-glycosides and kaempferol in *Bombyx mori* (Aiello et al., 2023). Since insects cannot manufacture these phytochemicals *de novo*, the discovery of copious amounts of rutin, quercetin, luteolin, apigenin and kaempferol in the two caterpillars suggest their possible sequestration from the leaves of mopane trees or synthesis through sclerotization (Nino et al., 2021). Plants are known to ubiquitously and vastly accumulate such metabolites for defense against herbivory and tolerance to harsh environmental conditions (Kwiri et al., 2020). The ability of polyphenolic compounds to act as singlet oxygen agents, reducing agents and hydrogen donors renders these caterpillars as source of bioactive antioxidants with diverse pharmacological benefits such as anti‐inflammatory, antimicrobial, anticancer, and insulin regulators, antibacterial inhibitors of the pancreatic lipase enzyme, and glycaemic inhibitors (Kunatsa et al., 2020; Roos and van Huis, 2017). The fact that Vanqa et al. (2022) evidenced scavenging activities and reducing power in the flours of *G. belina, Hermetia illucens* and *Macrotermes subhylanus* after drying and grinding demonstrates the possibility of insects conferring such health benefits even after processing. The detection of cholesterols and phytosterols in *G. belina* and *G. maja* resonates with other studies that found cholesterol, campesterol, stigmasterol and β-sitosterol in other phytophagous edible insects; *Zophobas morio* and *Tenebrio molitor, I. belina* and *Macrotermes bellicosus* (Kouřimská and Adámková, 2016), *Ruspolia differens* (Ochieng et al., 2022) and *Schistocerca gregaria* (Cheseto et al., 2015). Insects, unlike plants and vertebrates, are unable to biosynthesize such steroid compounds and therefore relies on plant-based dietary outsourcing to satisfy their metabolic requirements (van Huis et al., 2021). This is apparent in Table 7 where four phytosterols were also detected in the mopane tree leaves which the monophagous caterpillars in this study foraged on. Sterols are crucial compounds that serve intricate physiological roles such as cellular membrane stabilization and enzyme precursors, hence indispensable in the insects (Jing et al., 2012). Despite its adverse effects on health, cholesterol has been reported the most abundant steroid compound in insects (Papastavropoulou et al., 2022). The detection of stigmastan-3,5-diene, desmosterol, β-sitosterol and campesterol in this study indicates that these caterpillars may provide phytosterol-rich diet associated with retardation of cholesterol and plasma low density lipid-cholesterol intestinal absorption (Sabolová et al., 2016).

## 5 Conclusion

Nutritional assessment of wildly acquired *Gonimbrasia belina* and *Gynanisa maja* yielded excellent results of great nutritional relevance. The higher protein contents compared to conventional sources shows that the insects are cheap sources of proteins, that can contribute to alleviation of malnutrition cases among the local residents. Due to the high levels of limiting amino acids such as lysine and methionine, the caterpillars can be regarded as sources of proteins of considerable biological value. The presence of vitamins, ω−3 fatty acids, and copious levels of minerals (zinc, iron and calcium) characterized by rampant deficiencies implies that they are healthier food sources capable of combating hidden hunger. The detection of phytosterols and flavonoids shows that the mopane caterpillars are rich in bioactive compounds of known therapeutic value to humans. Summarily, the mopane worms can be regarded as complete novel food source, qualifying for nourishment of consumers with all the essential nutritional elements. However, stern measures and policies should be instituted to protect the destruction of mopane woodlands as well as promoting commercialization of this precious commodity for a sustainable utilization.

## Ethical statement

This research work did not embody experimentation on animals. This research was approved by the Institutional Animal Care and Use Committee (IACUC) of Kenya Agricultural and Livestock Research Organization (KALRO)-Veterinary Science Research Institute (VSRI); Muguga North upon adherence to all provisions vetted under and coded: KALRO-VSRI/IACUC028/16,032,022.

## Ethical statement - Studies in humans and animals

According to strict adherence to all the provisions provided by the Institutional Animal Care and Use Committee (IACUC) of Kenya Agricultural and Livestock Research Organization (KALRO)-Veterinary Science Research Institute (VSRI) [KALRO-VSRI/IACUC028/16,032,022], Muguga North, this research work was approved for implementation. Furthermore, this study was assessed and ratified by the National Council for Science Technology and Innovation in Kenya (NACOSTI/P/21/8303) and the University of Nairobi, Nairobi, Kenya, which provides permission to undertake sample collection from the field, processing, identification and experimental research in the laboratory.

## Funding sources

We strongly acknowledge the financial support from Bill and 10.13039/100000865Melinda Gates Foundation (INV-032416); Novo Nordisk Foundation (RefIPro: NNF22SA0078466), 10.13039/501100000974Australian Centre for International Agricultural Research (ACIAR) (ProteinAfrica –Grant No: LS/2020/154), IKEA Foundation (G-2204–02144), 10.13039/501100000780European Commission (HORIZON 101060762 NESTLER and HORIZON 101136739 INNOECOFOOD), the 10.13039/100000877Rockefeller Foundation (WAVE-IN—Grant No: 2021 FOD 030); the Curt Bergfors Foundation Food Planet Prize Award; 10.13039/100007843Norwegian Agency for Development Cooperation [RAF–3058 KEN–18/0005]; the Swedish International Development Cooperation Agency (Sida); the Swiss Agency for Development and Cooperation (SDC); the German Federal Ministry for Economic Cooperation and Development (BMZ); the Australian Centre for International Agricultural Research (ACIAR); the Government of Norway; the Federal Democratic Republic of Ethiopia and the Government of the Republic of Kenya. *The funders had no role in study design, data collection and analysis, the decision to publish, or the preparation of the manuscript. The views expressed herein do not necessarily reflect the official opinion of the donors*.

## CRediT authorship contribution statement

**Chrysantus M. Tanga:** Data curation, Software, Supervision, Methodology, Investigation, Formal analysis, Validation, Conceptualization, Resources, Writing – original draft, Writing – review & editing, Visualization, Project administration, Funding acquisition. **Brian O. Ochieng:** Writing – review & editing, Visualization, Validation, Methodology, Investigation, Conceptualization. **Dennis Beesigamukama:** Writing – review & editing, Visualization, Formal analysis, Conceptualization. **Changeh J. Ghemoh:** Conceptualization, Investigation, Validation, Visualization, Writing – review & editing. **Cynthia Mudalungu:** Writing – review & editing, Visualization, Validation, Formal analysis, Conceptualization. **Xavier Cheseto:** Writing – review & editing, Software, Methodology, Formal analysis, Conceptualization. **Isaac M. Osuga:** Writing – review & editing, Visualization, Validation, Methodology, Investigation. **Sevgan Subramanian:** Writing – review & editing, Visualization, Validation, Resources, Project administration. **Segenet Kelemu:** Writing – review & editing, Visualization, Validation, Resources, Project administration, Funding acquisition.

## Declaration of competing interest

The authors declare that they have no known competing financial interests or personal relationships that could have appeared to influence the work reported in this paper.

## Data Availability

Data will be made available on request.

## Appendix Supplementary materials

Image, application 1
