# Supplementary material for: Edible caterpillars (Gonimbrasia belina and Gynanisa maja) as emerging source of nutrients and bioactive compounds
Source: Future Foods. 2024 Dec;10:None. doi: 10.1016/j.fufo.2024.100478 (PMC11649843; doi:10.1016/j.fufo.2024.100478)

**Supplementary Information**

Total ion chromatogram indicating the present sterols in the given samples.


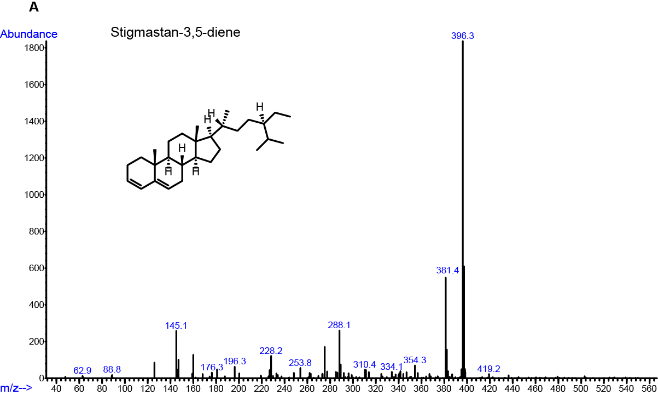


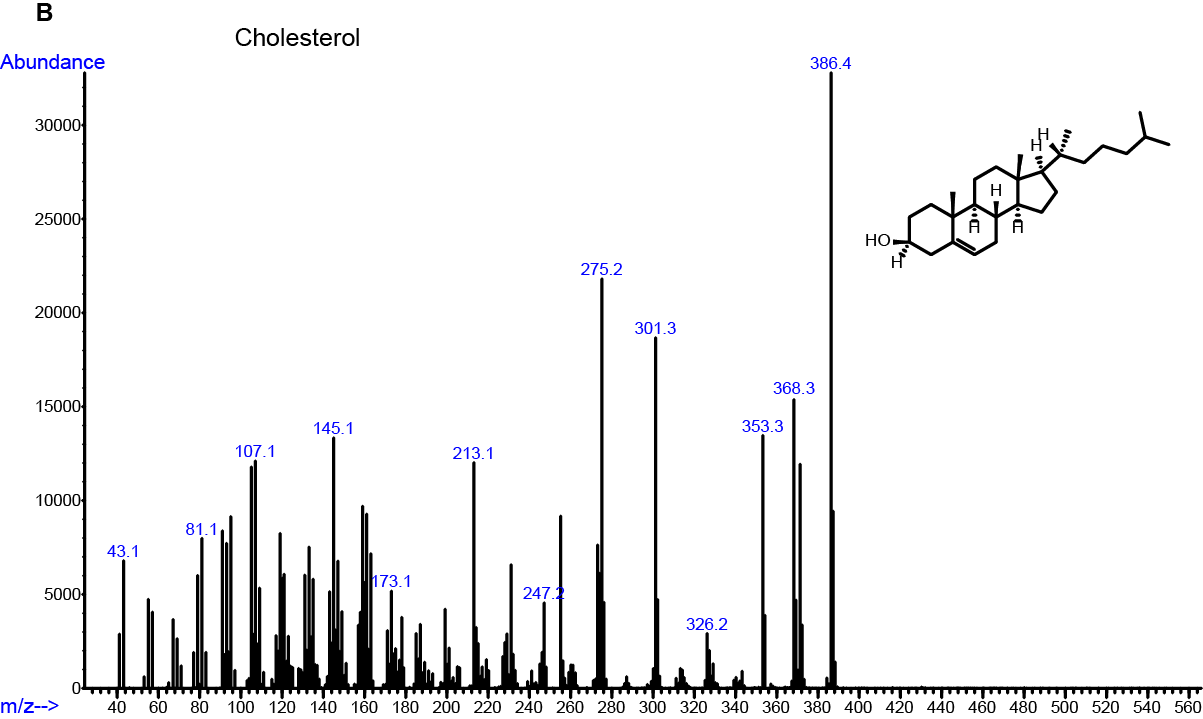


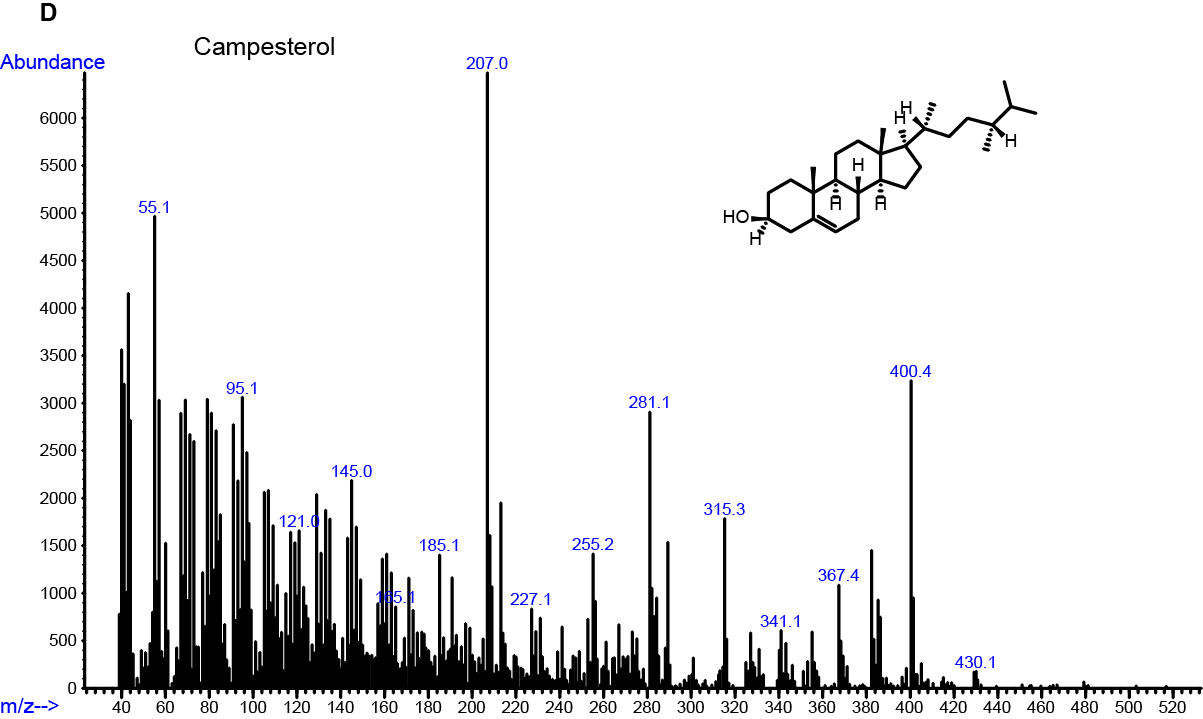

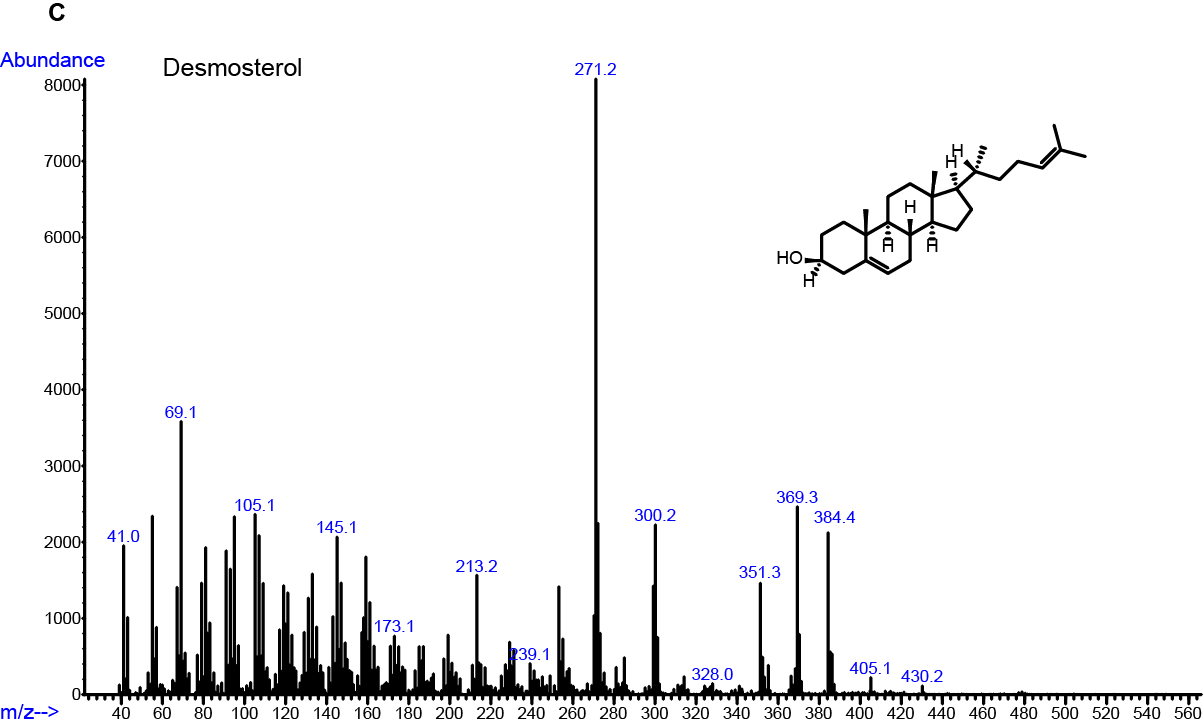


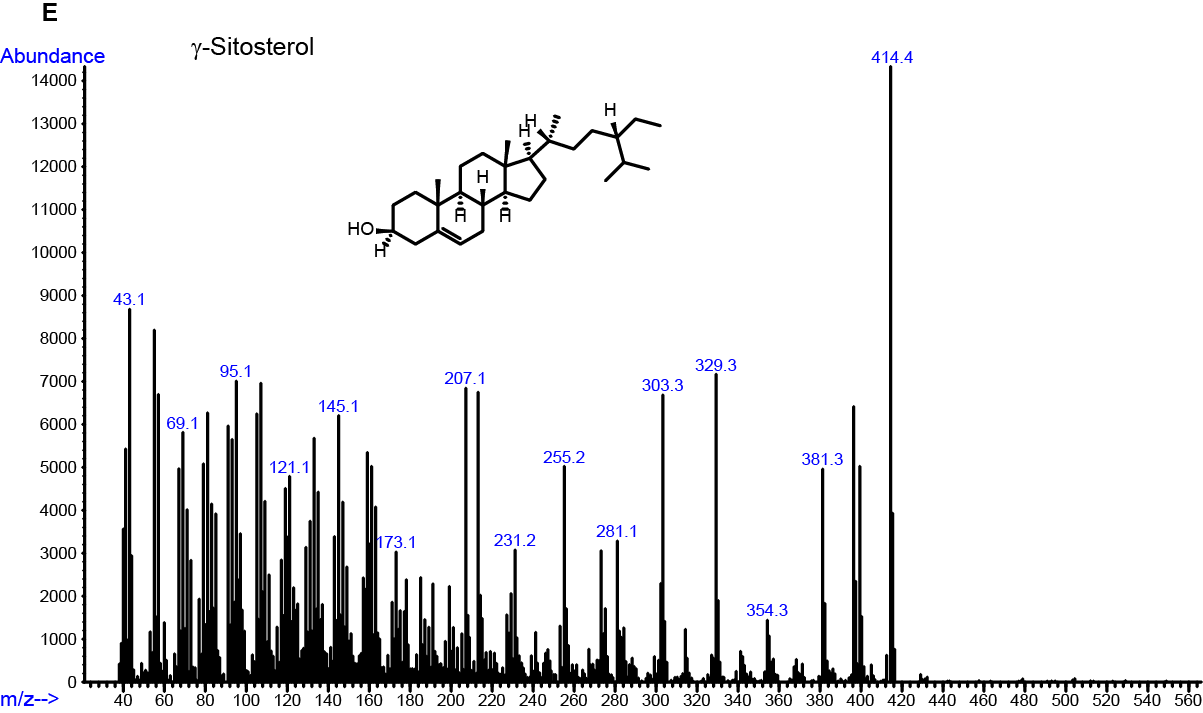


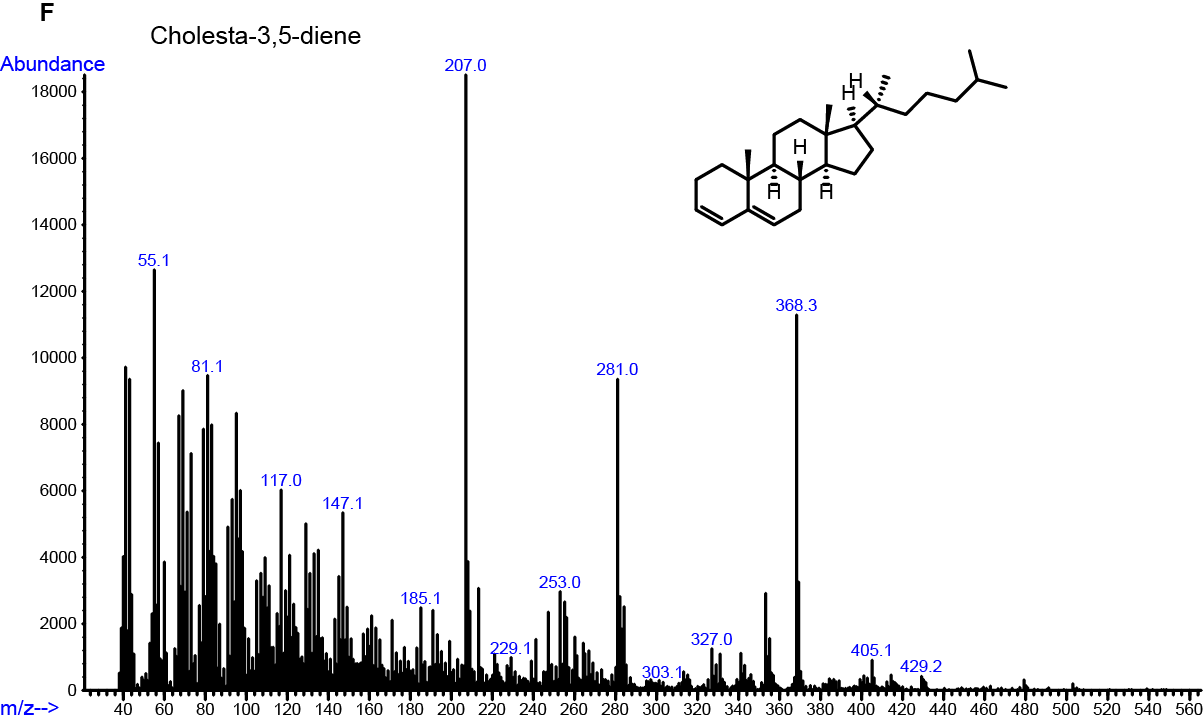


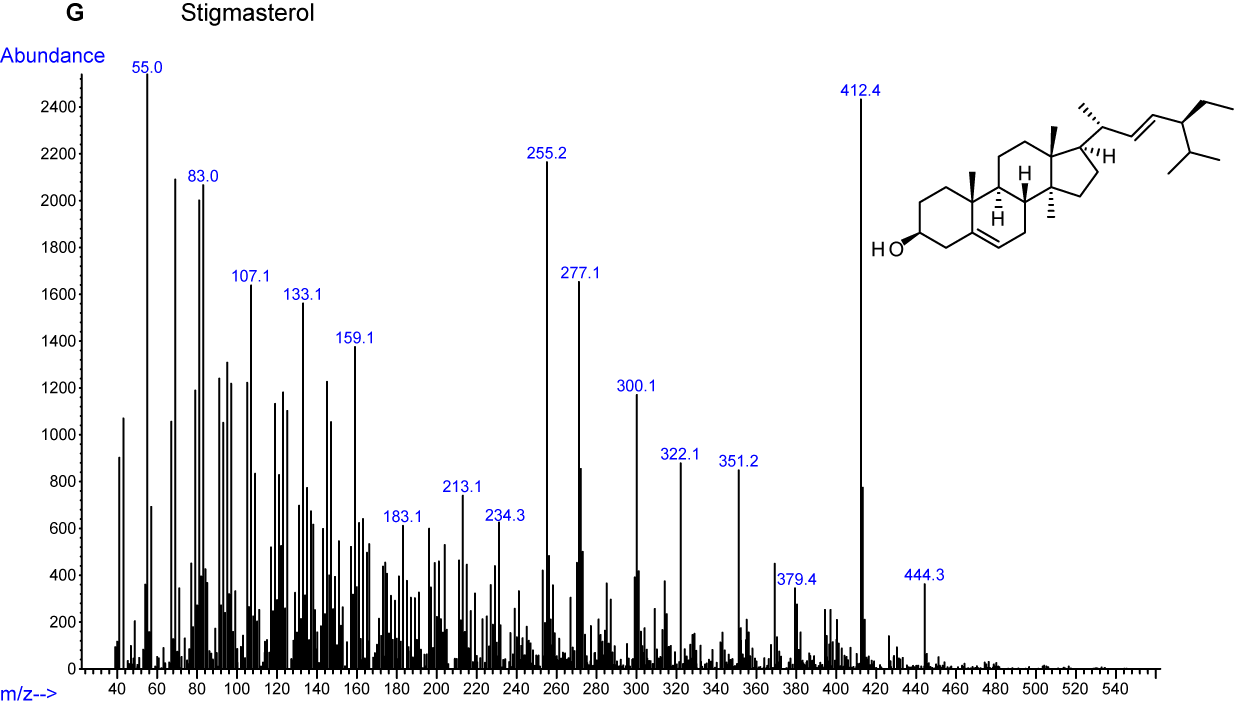


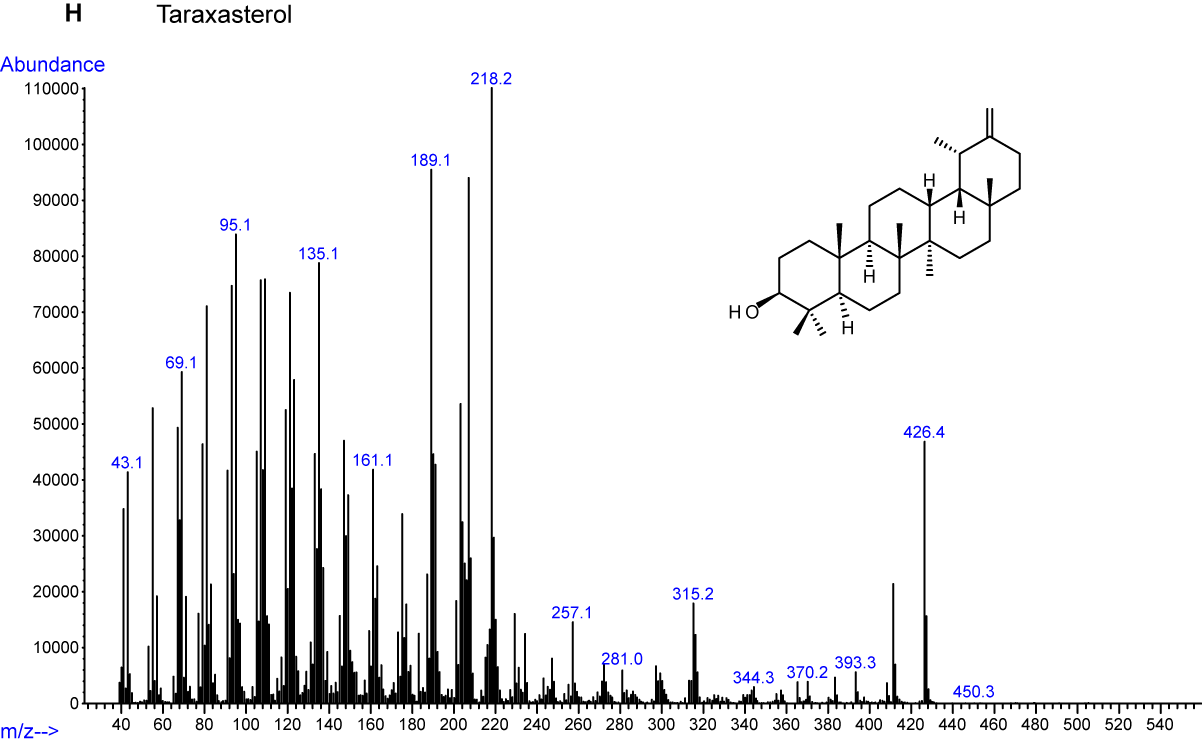

Supplement: Supplementary file 1 [file mmc1.docx]
